# Machine-Learning Modeling to Predict Hospital Readmission Following Discharge to Post-Acute Care Settings

**DOI:** 10.1093/geroni/igaa057.112

**Published:** 2020-12-16

**Authors:** Elizabeth Howard, John N Morris, Erez Schachter

**Affiliations:** 1 Boston College, Dover, Massachusetts, United States; 2 Hebrew SeniorLife, Boston, Massachusetts, United States; 3 Profility, Boston, Massachusetts, United States

## Abstract

Increased attention to post-acute care (PAC) settings and available services to meet patients’ needs following acute hospital discharge is needed as these settings are being utilized increasingly in models of care delivery. The primary purpose was to generate a model to identify the most predictive factors relevant to hospital readmission within 90 days following discharge to one of three types of PAC sites: home with home care services (HC), skilled nursing facility (SNF), in-patient rehabilitation facility (IRF). Specific aims were to (1) examine number and characteristics of older adults discharged to the 3 PAC sites; (2) compare 90 day hospital readmission rate across sites and acuity level; and (3) examine assessment items across population and subgroups to identify variables most predictive of hospital readmission. 2015 assessment data from 3,592,995 Medicare beneficiaries were analyzed representing 1,536,908 from SNFs, 306,878 from IRFs, and 1,749,209 receiving HC services. Total sample 90-day readmission was 25.8 % . Patients discharged to IRF had lowest readmission rate (23.34%), and those receiving HC services had highest readmission rate (29.34%). Creation of risk subgroups however, revealed alternative outcomes. Among all patients in the low, intermediate and high risk groups, the lowest readmission rates occurred among SNF patients. Factor analysis of assessment variables indicated bladder and bowel incontinence and functional limitations were the most distinguishing factors between the very low and very high risk subgroups.

